# Impact of free newborn care service package on out of pocket expenditure‐evidence from a multicentric study in Nepal

**DOI:** 10.1186/s12913-021-06125-9

**Published:** 2021-02-08

**Authors:** Avinash K Sunny, Omkar Basnet, Ankit Acharya, Prajwal Poudel, Mats Malqvist, Ashish KC

**Affiliations:** 1 Golden Community, Lalitpur, Nepal; 2 Ministry of Health and Population, Kathmandu, Nepal; 3 Department of Women’s and Children’s Health, Uppsala University, Dag Hammarskjölds väg 14B, Uppsala, Sweden

**Keywords:** Free Newborn Care Program, Impact, Out of Pocket Expenditure, Sick newborn, Nepal

## Abstract

**Background:**

Sustainable Development Goal *(*SDG) aspires to improve universal health coverage through reduction of Out of Pocket Expenditure (OOPE) and improving the quality of care. In the last two decades, there have been several efforts to reduce the OOPE for maternal and newborn care. In this paper, we evaluate the change in the OOPE for treatment of sick newborn at hospital before and after implementation of a free newborn care (FNC) program in hospitals of Nepal.

**Methods:**

Ministry of Health and Population implemented a free newborn care program which reimbursed the cost of treatment for all sick newborns admitted in public hospitals in Nepal from November 2017. We conducted this pre-post quasi-experimental study with four months of pre-implementation and 12 months of post-implementation of the program in 12 hospitals of Nepal. Logistic regression analysis was conducted for categorical variables and Mann-Whitney test was applied for continuous variables to determine statistically significant differences between pre- and post- intervention period.

**Results:**

A total of 353 sick newborns were admitted into these hospitals before implementation of the FNC program while 1122 sick newborns were admitted after the implementation. Before implementation, 17 % of mothers paid for sick newborn care while after implementation 15.3 % mothers (*p*-value = 0.59) paid for care. The OOPE for treatment of sick newborn at hospital before implementation was Mean ± SD: US dollar 14.3 + 12.1 and after implementation was Mean ± SD: USD 13.0 ± 9.6 (*p*-value = 0.71). There were no significant differences in neonatal morbidity after the implementation of the FNC program. The stay in a hospital bed (in days) decreased after the implementation of FNC program (*p*-value < 0.001) while the cost for medicine increased (*p*-value = 0.02). The duration of hospital stay (in days) of sick newborns significantly decreased for Hypoxic Ischemic Encephalopathy (HIE) (*p*-value = 0.04) and neonatal sepsis (*p*-value < 0.001) after the FNC program was implemented.

**Conclusions:**

We found no change in the OOPE for sick newborn care following implementation of the FNC Program. There is a need to revisit the FNC program by the type of morbidity and duration of stay. Further studies will be required to explore the health system adequacy to implement such programs in hospitals of Nepal.

**Trial registration:**

ISRCTN- 30829654, Registered on May 02, 2017.

## Background

One of the core principles of the Sustainable Development Goals (SDGs) is to leave no one behind in terms of accessing care [1]. This core principle provides impetus for universal health coverage (UHC)[2]. There has been a surge to introduce health financing schemes targeted for mothers and children in low-income settings where the financial burden is a major determinant to access care at health facilities[3]. In essence of these efforts for reducing the financial burden of travel and treatment expenses, a Maternity Incentive Scheme (MIS) was implemented in Nepal in 2005 which was further expanded to provide free delivery services in 2009[4].

One of the key barriers towards the achievement of UHC is out of pocket expenditure (OOPE), defined as direct payment for the cost of care[5, 6]. In order to mitigate such barriers to ensure UHC, there is a need to design effective interventions along with evidences and a financing strategy[7]. This is important as OOPE in many developing countries accounts for almost three-quarters or more of total expenditure on health[8–10]. Examples of financing schemes range from providing cash payments to mothers and families at the time of admission, voucher schemes during antenatal care, and reimbursing the cost of care at the health facilities[11–13]. Despite these efforts and investments by the global community to address concerns over high OOPE, the inequity gap for utilizing health facilities during childbirth has further widened in the last decade[14].

OOPE for sick newborn care can cause households to suffer catastrophic expenditures which can lead them into poverty[15]. Women from low socio-economic backgrounds who have to make direct payment for obstetric and neonatal complications are more vulnerable to catastrophic expenditures[16][17].

With increase in utilization of maternal and newborn care in last 20 years and with more than 60 % of deliveries now taking place in health facility, there is a need for specialized care for sick newborn in Nepal[18]. However, sick newborns who require specialized care and require longer duration of stay have implication of cost of care, usually overburdening parents for out of pocket expenditure [19, 20]. As a result, use of specialized care is limited due to the unbearable financial expenses required[21, 22]. To address this barrier, the Government of Nepal introduced a free newborn care (FNC) service program in public hospitals in 2016[23]. The scheme aims to align with pre-existing free health care scheme to provide subsidy for drugs, laboratory diagnosis and bed charges [23]. The FNC program is a financing scheme to provide subsidy for treatment for all sick newborns. The financing scheme aims to take no cost for treatment of sick newborn admitted in the hospital (admission charge, bed charge, laboratory diagnosis, drug and doctor fee).

As there is limited evidence on the impact of the free newborn care program on OOPE, this study was conducted to assess the impact of the FNC program introduced in Nepal by comparing OOPE of sick newborn treatment in hospital (admission charge, drugs, laboratory diagnosis, doctors fee and bed charge) before and after implementation in 12 public hospitals.

## Method

A pre-post quasi-experimental study, nested within a large stepped-wedged randomized control trial to evaluate the effectiveness of quality improvement interventions in 12 public hospitals of Nepal[24], was conducted among all births occurring in these hospitals between the 1 July 2017 and the 17 October 2018 with first four months as pre-intervention period and remaining twelve months as intervention period.

Based on the readiness of health facilities, newborn care in health facilities is classified into three levels- primary or basic neonatal level care at primary health care centers (level 1), secondary level care at special newborn care unit (SNCU) (level 2) and tertiary level care at neonatal intensive care unit (NICU) (level 3) [25]. As of November 2017, the FNC service package was implemented in all the public hospitals across the country. Department of Health Services, Child Health Division reimbursed the costs to public hospitals which provide free newborn care package based on the level of newborn care. The amount of reimbursement to each hospital per sick newborn is given based on the package of free newborn implemented (Table 1). The hospital which implement package A receive 9.6 USD per sick newborn admitted. The package A consists of basic sick newborn care. The hospital which implement package B receive 19.2 USD per sick newborn admitted. The package B consists of specialized newborn care. The hospital which implement package C receive 48.0 USD per sick newborn admitted. The package C consists of neonatal intensive care.

**Table 1:** Free Newborn Care Service Program

Package	Services	Level of newborn care	Reimbursement United States Dollar per sick newborn admitted
“A”	• Medicines- Antibiotics, dextrose, IV Canula, etc. • Laboratory services including blood testing • Oxygen Supply by hood box /nasal prong • X-ray / USG	Basic Sick Newborn Care	9.6
“B”	• Photo therapy • Laboratory Services- Blood culture, RFT (Sodium, Potassium, Urea creatinine), Serum calcium • Lumbar Puncture and CSF Analysis • Medicine- Dopamine, Dobutamine, Phenobarbitone, Phenytoin, Midazolam, calcium Gluconate, Aminophylene • Bubble CPAP (Continuous Positive Airway Pressure)	Specialised Sick Newborn Care	19.2
“C”	• NICU Admission (Must) • NICU bedside Ultrasonography (USG) • NICU bedside Portable X-Ray •Lab: ABG, Magnesium, Chloride, Serum Osmolarity, Urine Specific Gravity, Urine Electrolyte •Double Volume Exchange Transfusion, Blood transfusion • Medicine: Caffeine • Mechanical Ventilation	Neonatal Intensive Care Service	48.0

### Setting

Study was conducted in the twelve government-funded hospitals where referral care for mothers and newborns are provided. These hospitals varied in terms of the utilization of care, geographical location, religious ethnicity, language. Four of the hospitals had more than 8,000 deliveries a year, four hospitals 3,000-8000 deliveries a year, and the remaining four hospitals had 1,000-3000 deliveries a year. The low volume hospitals (Bardiya, Pyuthan, Nuwakot, and Nawalparasi) did not have specialized care services for sick newborn. The high-volume hospitals (Koshi Zonal, Bharatpur, Lumbini Zonal, and Bheri Zonal) and medium-volume hospitals (Western Regional, Rapti Sub-Regional, Mid-Western Regional, and Seti Zonal) provided specialized newborn care services. The SNCUs s were led by the pediatricians while in low volume hospitals, sick newborns were managed by medical doctors at the pediatric unit. In the sick newborn care units, management of sick newborns was done by medical doctors with 24 hours care from nursing staff.

### Participants

All babies delivered in the study period who were admitted for sick newborn care were included in this study. Births with missing data on the cost of care were excluded from the analyses.

### Variables and outcomes

**Out-of-pocket expenditure (OOPE**) was defined as a fee made by an individual for a consultation with a health professional, an investigation or procedure, medicines, supplies and laboratory tests.

**Neonatal morbidity**:  Sick newborns were classified having any of the following diagnoses[26]:

- Hypoxic Ischemic Encephalopathy (HIE): Syndrome of abnormal neurological behavior in the neonate, which is frequently associated with multi-system dysfunction and follows severe injury before or during delivery.
- Neonatal sepsis (NNS): Clinical signs of severe bacterial infection, with a blood culture positive for a pathogenic organism.
- Hyperbilirubinemia (HBL): Babies with total Serum Bilirubin (TSB) increasing by >5 mg/dl/day or 0.5 mg/dl/hour, TSB>15 mg/dl, conjugated serum bilirubin > 2 mg/dl.
- Meconium aspiration syndrome (MAS): Breathing problems that a newborn baby may have when there are no other causes, and the baby has passed meconium (stool) into the amniotic fluid during labour or delivery.
- Respiratory Distress Syndrome (RDS): Neonate with signs of respiratory distress-cyanosis, tachypnoea (>60/min, shallow, rapid), grunting (delayed expiration maintains Functional residual capacity), retraction (Subcostal, sub-sternal, intercostal), flaring (50% airway resist in nose& pharynx).

Others included low birth weight, shoulder dystocia, hypoglycemia, congenital malformation, etc.

### Data collection and management

Data were collected through an independent data collection team established in all the hospitals. The data collectors extracted obstetric and neonatal information from from the maternity registers and medical records using an extraction form. Information of socio-demographic variables and and OOPE were collected by the data collectors at through semi-structured interviews with mothers before discharge. All filled up forms were then assessed by a data coordinator on completeness and accuracy at each hospital. Data were then entered into the data base by the data entry and management team using the Census and Survey Processing System (CSPro).

### Statistical analysis

The cleaned data were imported into Statistical Package for Social Sciences (SPSS) version 23 for analysis. Descriptive statistics were presented with mean, standard deviation (SD), median, interquartile range (IQR), frequency and percentage. Logistic regression was used to compare background characteristics of the sick newborns and the Mann-Whitney test was applied for comparing the cost of care between the groups. Mann-Whitney U test was used to compare the difference of OOPE between two different time periods and the OOPE might not have been normally distributed. Logistic regression was used to explore the association between sick newborns and expenditure for services received. P-value < 0.05 was considered to be statistically significant. Missing data were excluded from the analyses.

### Ethical approval and consent

 Written informed consent was obtained from the mothers before inclusion in the study and confidentiality was maintained. The study was approved by Ethical Review Board of Nepal Health Research Council (reference number 26-2017).

## Results

**Fig. 1:**
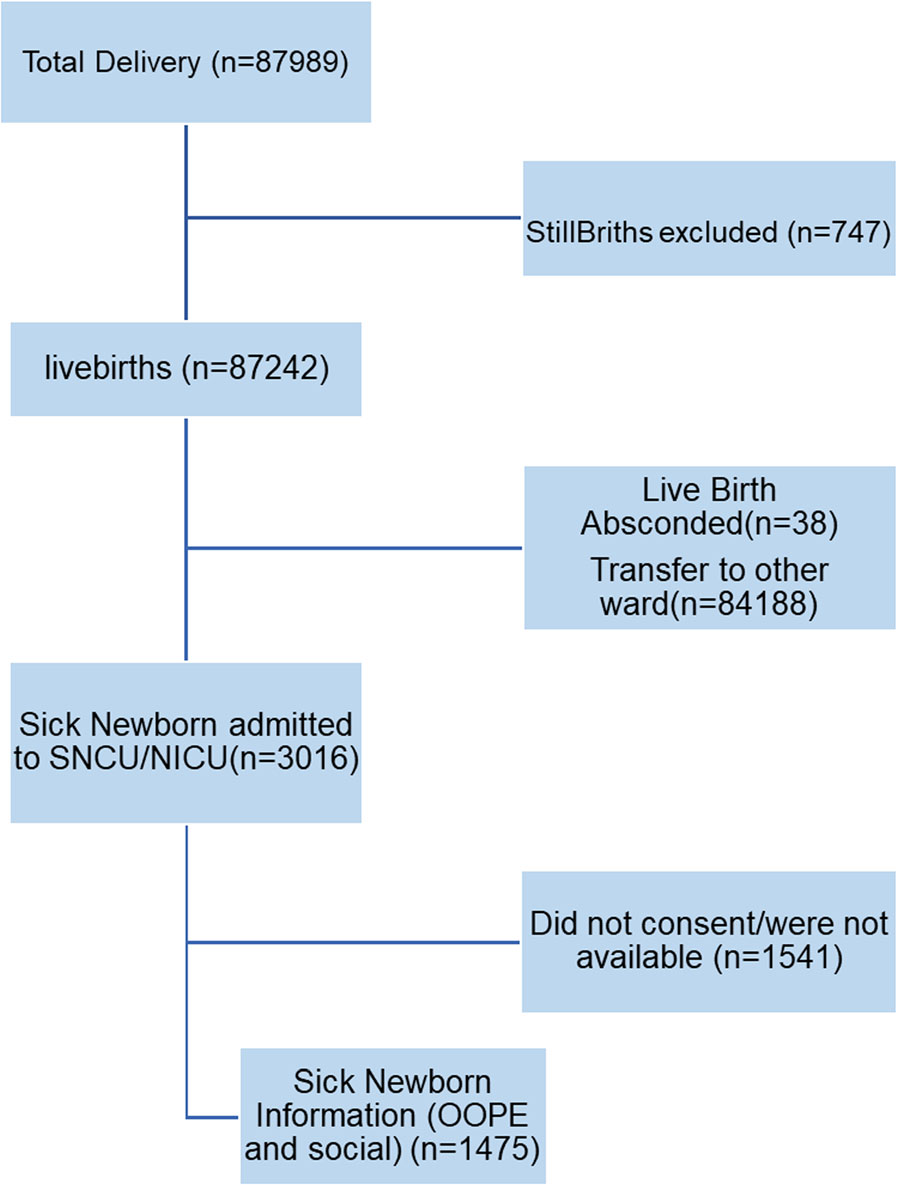
Study flow diagram of the participants

There was a total of 87,989 deliveries observed in the study period, out of which 87,242 were livebirths and 747 stillbirths. Among these livebirths, 3016 sick newborns were admitted into the special newborn care unit and neonatal intensive care unit. Out of these sick newborns we could obtain consent for out of pocket expenditure information for a total of 1475 sick newborns (Fig. 1).

A total of 353 sick newborns were admitted into these hospitals before free newborn care (FNC) program was implemented while 1122 sick newborns were admitted after the implementation. Sick newborns admitted in the hospital varied by various characteristics before and after the implementation of FNC program. The proportion of mothers of admitted sick newborn with basic education was higher after implementation of FNC program than before implementation (15.5 % vs. 10.5 %, *p* value-0.004). The proportion of mothers of admitted sick newborns from madeshi ethnic group was higher after implementation of FNC program than before implementation (8.4 % vs. 4.2 %, *p*-value-0.003). The proportion of mothers of admitted sick newborns from muslim ethnic group was higher after implementation of FNC program than before implementation (3.4 % vs. 1.7 %, *p*-value-0.03) The proportion of sick newborn born to multiparous mothers was lower after implementation of FNC program than before implementation (31.5 % vs. 42.5 %, *p*-value-0.01) (Table 2).

**Table 2:** Background Characteristics of the Sick Newborn

Variables	Before FNC (*n* = 353)	After FNC (*n* = 1122)	Total (*n* = 1475)	*p*-value
**Maternal age**	23.65 ± 4.01	24.09 ± 4.49	23.98 ± 4.38	
20–35	254 (72.0 %)	818 (72.9 %)	1072 (72.7 %)	
< 20	96 (27.2 %)	285 (25.4 %)	381 (25.8 %)	0.56
≥ 35	3 (0.8 %)	19 (1.7 %)	22 (1.5 %)	0.28
**Education**				
Secondary and above	266 (75.4 %)	717 (63.9 %)	983 (66.6 %)	
Illiterate	19 (5.4 %)	94 (8.4 %)	113 (7.7 %)	**0.02**
Literate	31 (8.8 %)	137 (12.2 %)	168 (11.4 %)	**0.02**
Basic education	37 (10.5 %)	174 (15.5 %)	211 (14.3 %)	**0.004**
**Ethnicity**				
Dalit	47 (13.3 %)	174 (15.5 %)	221 (15.0 %)	0.06
Janjati	102 (28.9 %)	308 (27.5 %)	410 (27.8 %)	0.18
Madhesi	15 (4.2 %)	94 (8.4 %)	109 (7.4 %)	**0.003**
Muslim	6 (1.7 %)	38 (3.4 %)	44 (3.0 %)	**0.03**
Chhetri/Brahmin	160 (45.3 %)	460 (41.0 %)	620 (42.0 %)	0.24
Others	23 (6.5 %)	48 (4.3 %)	71 (4.8 %)	
**Parity (previous birth)**				
Primipara (1)	126 (35.7 %)	422 (37.6 %)	548 (37.2 %)	
Nullipara (0)	77 (21.8 %)	347 (30.9 %)	424 (28.7 %)	0.07
Multipara (2–5)	150 (42.5 %)	353 (31.5 %)	503 (34.1 %)	**0.01**
**Neonatal Morbidity**				
HIE	89 (25.2 %)	275 (24.5 %)	364 (24.7 %)	0.54
NNS	114 (32.3 %)	310 (27.6 %)	424 (28.7 %)	0.14
HBL	11 (3.1 %)	31 (2.8 %)	42 (2.8 %)	0.60
MAS	21 (5.9 %)	82 (7.3 %)	103 (7.0 %)	0.61
RDS	6 (1.7 %)	42 (3.7 %)	48 (3.3 %)	0.11
Others	112 (31.7 %)	382 (34.0 %)	494 (33.5 %)	
**Birth Weight**				
2500–4000	241 (68.3 %)	770 (68.6 %)	1011 (68.5 %)	
< 2500	103 (29.2 %)	334 (29.8 %)	437 (29.6 %)	0.91
≥ 4000	9 (2.5 %)	18 (1.6 %)	27 (1.8 %)	0.26
**Gestational Age**				
37–42 weeks	211 (59.8 %)	680 (60.6 %)	891 (60.4 %)	
< 37 weeks	113 (32.0 %)	383 (34.1 %)	496 (33.6 %)	0.71
≥ 42 weeks	29 (8.2 %)	59 (5.3 %)	88 (6.0 %)	0.06

*cOR crude Odds Ratio

We compared the duration of stay (in days) of sick newborns by neonatal morbidity before (n-353) and after implementation (n-1122) of FNC. The duration of stay for babies with HIE after (3.3 ± 5.9 days) implementation of FNC was lesser than before (3.5 ± 3.9 days) implementation (*p* value = 0.04). The duration of stay for babies with neonatal sepsis after (3.7 ± 5.9 days) implementation of FNC was lesser (4.4 ± 4.2 days) than before implementation (*p* value < 0.001) (Table 3).

**Table 3:** Duration of Stay in days by Neonatal morbidity

Duration of stay	N (353)	Before Free Newborn Care		N (1122)	After Free Newborn Care		*p*-value
		Mean ± SD	Median (IQR)		Mean ± SD	Median (IQR)	
HIE	88	3.48 ± 3.86	2.50 (0–16)	269	3.28 ± 5.86	2 (0–38)	**0.04**
NNS	112	4.39 ± 4.22	4 (0–31)	306	3.71 ± 5.91	2 (0–39)	**< 0.001**
HBL	11	5.27 ± 5.15	3 (0–17)	31	5.19 ± 6.48	4 (0–35)	0.88
MAS	20	3.80 ± 3.76	3 (0–16)	82	3.60 ± 6.08	3 (0–38)	0.23
RDS	6	11.67 ± 12.70	7 (0–35)	41	3.17 ± 5.95	2 (0–36)	0.09
Others	112	4.53 ± 5.43	3 (0–38)	375	4.01 ± 6.83	2 (0–38)	**< 0.001**

Before implementation of FNC program, 17 % (60) mothers paid for sick newborn care services, while 15.3 % (172) mother paid for sick newborn care after implementation. After adjusting with maternal, obstetric and neonatal characteristics, there was no change in proportion of mothers paying for sick newborn before and after implementation of FNC program after adjusting for socio-demographic characteristics (aOR-0.91, 95 % CI, 0.65–1.28, p = 0.59). (Table 4)

**Table 4:** Association between those who paid for services before and after FNC implementation

Variables	Before FNC (*n* = 353)	After FNC (*n* = 1122)	Total (*n* = 1475)	cOR (95 % CI)	aOR (95 % C.I.)^a^
**Paid for services**					
No	293 (83.0 %)	950 (84.7 %)	1243(84.3 %)	Reference	Reference
Yes	60 (17 %)	172 (15.3 %)	232 (15.7 %)	1.13 (0.82–1.56)	0.91 (0.65–1.28)

Cox and Snell pseudo *R*-squared: 0.04; Nagelkerke pseudo *R*-squared: 0.06;

Hosmer and Lemeshow Test: x^2^(8) = 5.35, *p* = 0.72

^a^adjusted for maternal age, education, ethnicity, parity, neonatal morbidity, birth weight and gestational age

Among those who paid for the sick newborn care services (n-232), there were significant differences in the expenditure by type of service used before (n-60) and after the implementation (n-172) of the FNC program. The bed charge significantly decreased from USD 6.4 ± 6.5 to USD 3.1 ± 5.3 after implementation (*p*-value < 0.001), while cost for medicine increased from Mean ± SD USD 3.4 ± 10.8 to Mean ± SD NPR 4.4 ± 9.5 after implementation (*p*-value = 0.02). However, there was no significant difference in the OOPE (total service expenses) before (Mean ± SD: USD 14.4 + 12.1) and after (Mean ± SD: USD 13.0 ± 9.6) implementation (*p*-value = 0.71) (Table 5).

**Table 5:** OOPE by type of services for care among those who paid for the services

	Before FNC (N-60)	After FNC (N-172)	*p*-value *
**Admission charge**			
Mean ± SD	0.1 ± 0.5	0.14 ± 1.3	0.21
Median (IQR)	0(0-3.8)	0(0-14.4)	
**Bed charge**			
Mean ± SD	6.4 ± 6.5	3.1 ± 5.3	**< 0.001**
Median (IQR)	450(0–24.0)	0(0–24.0)	
**Laboratory diagnosis**			
Mean ± SD	2.5 ± 3.2	3.4 ± 4.2	0.55
Median (IQR)	2.4 (0-13.4)	2.4(0-15.5)	
**Doctor fees**			
Mean ± SD	1.9 ± 0.1	1.9 ± 0.2	0.66
Median (IQR)	1.9 (0.9–1.9)	1.9(1.1–4.3)	
**Medicines**			
Mean ± SD	3.4 ± 10.8	4.3 ± 9.5	**0.02**
Median (IQR)	0(0-57.6)	0(0-96.1)	
**Total Service Expense**			
Mean ± SD	14.5 + 12.1	13.0 ± 9.6	0.71
Median (IQR)	12.0(3.8–67.7)	11.9(2.1–98.0)	

*Mann-Whitney test

Similarly, the cost of drugs and diagnostics for sick newborns with major neonatal morbidity did not show significant difference after the implementation except for that of other neonatal morbidity which increased from USD 4.0 ± 4.9 to USD 9.8 ± 13.2 (p-value = 0.007) (Table 6).

**Table 6:** Cost of care in US dollars by Neonatal morbidity

Drugs and Diagnostics	N (60)	Before Free Newborn Care		N (172)	After Free Newborn Care		*p*-value
		Mean ± SD	Median (IQR)		Mean ± SD	Median (IQR)	
HIE	7	3.2 ± 2.7	2.4 (0-8.6)	34	7.5 ± 7.5	6.5 (0-38.4)	0.10
NNS	24	6.7 ± 13.0	2.4 (0-64.4)	39	6.1 ± 6.2	2.4 (0-25.9)	0.32
HBL	6	2.9 ± 1.9	2.4 (0-5.3)	8	3.1 ± 2.8	2.4 (0-9.6)	0.89
MAS	6	13.7 ± 23.1	4.1 (0–60.0)	21	8.1 ± 7.5	9.6 (0-34.8)	0.81
RDS	2	7.9 ± 7.8	7.9 (2.4–13.4)	10	6.1 ± 3.8	6.2 (0–10.0)	0.66
Others	15	4.0 ± 4.9	2.4 (0-17.3)	60	9.8 ± 13.2	7.7 (0–96.0)	**0.007**

## Discussion

The FNC program was implemented with the intention to overcome the burden of out of pocket expenditure on sick newborn treatment in the hospital. However, we found no significant change in the OOPE for treatment of sick newborn in the hospital before and after implementation of FNC. Though total expense among those who paid for the services received did not change significantly, there was a significant decrease in bed charge and significant increase in medicinal cost after the implementation of FNC program. This reflects that medicines may have been used not as per protocol.

Sick newborns admitted into the hospitals varied by various characteristics such as maternal education, ethnicity, parity and neonatal morbidity. After implementation of FNC, the use of sick newborn care was higher among mothers who were illiterate, literate and had basic education than before implementation. In contrast to this finding, various studies have shown that utilization of maternal and newborn care services increases with higher educational level due to higher health literacy and understanding of service utilization[27, 28].

After the implementation FNC, the proportion of sick newborns from Madhesi and Muslim ethnic groups was higher than before implementation. Also, the proportion of sick newborns admitted in the hospital from multi-parous mother was higher after FNC than before implementation. This finding is similar with another study conducted in Nepal which reported that primiparous mothers have significant increment in neonatal care utilization in comparison to those of multiparous mothers[29].

Comparing the sick newborns with different neonatal morbidities, the duration of stay significantly decreased for HIE and neonatal sepsis (NNS) after the FNC program was implemented. Average duration of stay in this study significantly decreased for NNS accounting for the highest number of admissions from 4.39 ± 4.22 days to 3.71 ± 5.91 days after the implementation of FNC program. Similarly, it decreased from 3.48 ± 3.86 to 3.28 ± 5.86 for HIE. This could be due to unavailability of medicines or diagnostics leading to an increase in the number of referrals from these public hospitals to other centers. A study conducted in Enugu, southeast Nigeria found that NNS caused the highest number of admissions, accounting for 61 % of all neonatal admissions during the period under review, with a mean duration of hospital stay of 15.3 ± 9.6 days and a wide range of 4–50 days. The average duration of stay in tertiary public hospitals has been reported to be higher resulting in increased cost of neonatal care[19]. The lower duration of stay in this study for sick newborns with HIE and neonatal sepsis may suggest that costs exceeded the total cost of reimbursement from the FNC program.

Similarly, the cost of drugs and diagnostics for sick newborns with major neonatal morbidity (HIE, NNS, HBL, MAS, RDS) did not show significant differences after the implementation. However, studies have reported that the cost of sick newborn care is directly proportional to type of diagnosis[19, 30]. Major neonatal morbidities like HIE and neonatal sepsis requires tailored management with varying medications for sick newborns, however, these are not reimbursed through the FNC program. The implementation of FNC program did not result in significant amount of differences in out-of-pocket expenditure for newborn care. The coverage of this package is not exhaustive to include diagnostics like PT/INR (Prothrombin Time/International Normalised Ratio), reimbursement for blood products (like packed RBC, Fresh-Frozen Plasma, Platelet-Rich Plasma and whole blood), baby diapers and surfactants for Respiratory Distress Syndrome (RDS) which has to be purchased out-of-pocket in many cases. There is a need for the evaluation of the program for better implication of the free sick newborn policy.

There are some limitations in this study. This study did not assess the expenditure for sick newborns after referral which may reflect the direct impact of FNC program. This study may be exposed to recall bias relating to the mothers’ recall of expenses paid for the services received. Some information could have resulted in social desirability bias as there is a wide range in terms of expenses reported. We assume that they may have sometimes reported information based on their interpretation which is socially relevant.

## Conclusions

There was no change in OOPE for sick newborns after the implementation of FNC program. The duration of hospital stays of sick newborns for various neonatal morbidities significantly decreased after the FNC program was implemented. This might imply that the OOPE per day might have increased. The cost of drugs and diagnostics in hospitals did not vary for various morbidities in sick newborns after the implementation of the FNC program. There is a need for further studies to explore the health system adequacy to implement financing strategies for free newborn care.

## Data Availability

The datasets used and/or analysed during the current study are available from the corresponding author on reasonable request.

## Abbreviations

aOR: Adjusted Odds Ratio
cOR: Crude Odds Ratio
CSPro: Census and Survey Processing System
FNC: Free Newborn Care Program
HBL: Hyperbilirubinemia
HIE: Hypoxic Ischemic Encephalopathy
IQR: Interquartile range
MAS: Meconium Aspiration Syndrome
MIS: Maternal Incentive Scheme
NICU: Neonatal Intensive Care Unit
NNS: Neonatal Sepsis
OOPE: Out of Pocket Expenditure
PT/INR: Prothrombin Time/International Normalised Ratio
RDS: Respiratory Distress Syndrome
SD: Standard deviation
SNCU: Specialized Newborn Care Unit
SPSS: SPSS-Statistical Package for Social Sciences
UHC: Universal Health Coverage
